# Human Cytomegalovirus Envelope Protein gpUL132 Regulates Infectious Virus Production through Formation of the Viral Assembly Compartment

**DOI:** 10.1128/mBio.02044-20

**Published:** 2020-09-29

**Authors:** Hui Wu, Barbara Kropff, Michael Mach, William J. Britt

**Affiliations:** a Department of Microbiology, School of Medicine, University of Alabama in Birmingham, Birmingham, Alabama, USA; b Virologisches Institut, Klinische und Molekulare Virologie, Friedrich-Alexander-Universität Erlangen-Nürnberg, Erlangen, Germany; c Department of Pediatrics, University of Alabama in Birmingham School of Medicine, Birmingham, Alabama, USA; Princeton University

**Keywords:** human cytomegalovirus, viral assembly compartment, virion envelope protein, virus glycoproteins

## Abstract

The human cytomegalovirus (HCMV) UL132 open reading frame encodes a 270-amino-acid type I envelope glycoprotein, gpUL132. The deletion of UL132 (ΔUL132) from the HCMV genome results in a pronounced deficit in virus yield, with an approximately 2-log decrease in the production of infectious virus compared to the wild-type (WT) virus. Characterization of the ΔUL132 mutant virus indicated that it was less infectious with a high particle-to-infectious unit ratio and an altered composition of virion proteins compared to the WT virus. In addition, the viral assembly compartment (AC) failed to form in cells infected with the ΔUL132 mutant virus. The expression of gpUL132 in *trans* rescued the defects in the morphogenesis of the AC in cells infected with the ΔUL132 mutant virus and in infectious virus production. Furthermore, using cell lines expressing chimeric proteins, we demonstrated that the cytosolic domain of gpUL132 was sufficient to rescue AC formation and WT levels of virus production. Progeny virions from ΔUL132-infected cells expressing the cytosolic domain of gpUL132 exhibited particle-to-infectious unit ratios similar to those of the WT virus. Together, our findings argue that gpUL132 is essential for HCMV AC formation and the efficient production of infectious particles, thus highlighting the importance of this envelope protein for the virus-induced reorganization of intracellular membranes and AC formation in the assembly of infectious virus.

## Abstract (precis)

Following infection of permissive cells, human cytomegalovirus (HCMV) induces the reorganization of intracellular membranes resulting in the formation of a distinctive membranous compartment in the cytoplasm of infected cells. This compartment has been designated the viral assembly compartment (AC) and is thought to be a site for cytoplasmic virion assembly and envelopment. In this study, we have demonstrated that a single virion envelope glycoprotein is essential for AC formation in infected cells, and in its absence, there is a significant decrease in the production of infectious virions. These findings are consistent with those from other studies that have demonstrated the importance of host cell proteins in the formation of the AC and demonstrate a critical role of a single virion protein in AC formation and the efficient assembly of infectious virus.

## INTRODUCTION

Worldwide, human cytomegalovirus (HCMV) infects between 50% and 80% of the population. In immunocompetent hosts, HCMV rarely causes clinical symptoms or disease. Nonetheless, HCMV is an important cause of morbidity and mortality in immunocompromised hosts, particularly in organ transplant recipients. HCMV is the most frequent virus that is transmitted *in utero* to the developing fetus. Intrauterine transmission of HCMV can result in long-term neurodevelopmental sequelae in infected infants and children, including hearing loss. HCMV is composed of three distinct structures, a capsid containing the double-stranded DNA genome, a tegument layer, and an outermost envelope layer that is predicted to contain more than 50 viral glycoproteins. The 235-kb genome of HCMV encodes more than 240 open reading frames, a large number of microRNAs, and a number of long noncoding RNAs. Several virion glycoproteins have been shown to be essential for virus infectivity, including the conserved core glycoproteins present in most herpesviruses, gB, gH, gL, and gM. Glycoprotein B is thought to be the HCMV fusogen, and when complexed with gH/gL, it can result in cell fusion (1, 2). In addition, glycoproteins unique to HCMV, such as gO, a component of the gH/gL/gO trimer, are required for both cell-free and cell-to-cell spread of the virus (3–5). Another glycoprotein complex unique to HCMV is a pentameric complex consisting of gH/gL combined with products of the UL129-131A open reading frames, gH/gL/UL129-131A, that has been shown to be required for efficient infection of myeloid cells, endothelial cells, and epithelial cells but dispensable for infection of fibroblasts (3, 6–10). In contrast to these well-studied glycoproteins, a large number of virus-encoded glycoproteins, including some virion structural proteins, are not essential for infectivity *in vitro*, and as a result, their role in the replicative cycle of HCMV is less well understood.

UL132 encodes a type I membrane glycoprotein, gpUL132, that is relatively abundant in the virion envelope (11). Although HCMV genomic sequences adjacent to UL132 are frequently deleted in HCMV strains that have been passaged extensively *in vitro* in human fibroblast cells, UL132 is conserved across different HCMV strains regardless of their passage history (12). Interestingly, UL132 does not have a homolog in alpha- and gammaherpesviruses. UL132 is not absolutely essential for virus replication *in vitro*, but the yield of infectious viruses from cells infected with a recombinant virus with a deletion of UL132 is about 100-fold lower than that from wild-type (WT) virus-infected cells (13). The mechanism that accounts for the decrease in virus yield in ΔUL132 HCMV-infected cells has not been defined, but interestingly, mutation of four endocytic motifs that are present in the cytosolic domain of gpUL132 has been shown to decrease the virus yield by nearly 100-fold, limit the incorporation of a mutant gpUL132 into the virion, and recapitulate several of the *in vitro* phenotypes of the gpUL132 deletion mutant virus (13, 14). These endocytic motifs in the cytosolic domain of gpUL132 have been shown to allow the efficient retrieval of gpUL132 from the plasma membrane by clathrin-dependent endocytosis and the incorporation of gpUL132 into the virion (14). Thus, gpUL132 clearly has been shown to play a significant role in the generation of infectious virions, yet a detailed understanding of its function in the replicative cycle of HCMV infectivity is lacking.

A well-described characteristic of HCMV-infected human fibroblast (HF) cells during lytic infection is the development of an enlarged reniform or kidney-shaped nucleus together with a juxtanuclear membranous structure rich in both host cell and virion proteins (15–17). The latter cytoplasmic structure in infected cells has been termed the assembly compartment (AC) or the virus assembly compartment (vAC) (17). The AC has been shown to be composed of HCMV virion proteins, including essential glycoproteins and tegument proteins, and an undetermined number of host cell proteins, including major components of the cellular secretory and endocytic systems (15, 18–20). The AC is positioned in proximity to the concavity of the reniform nucleus and localized to the microtubule organizing center (MTOC) of the infected cell (15, 21). Image analysis suggested that the AC is organized as a series of concentric accumulations of resident proteins of the endoplasmic reticulum (ER), *cis*-Golgi, *trans*-Golgi, and endocytic proteins that surround the membrane-associated virion proteins (15, 18, 20, 22, 23). The morphogenesis of the AC has been shown to be dependent on an as-yet-undefined number of host proteins that are thought to contribute to intracellular membrane organization and trafficking, such as HSP GRP78 (BiP), Grasp65, dynein, and Syntaxin 5 (24–29). In addition, virus-encoded microRNAs (vmiRNAs) that target components of the secretory and endocytic pathways have been shown to contribute to the formation of the AC (30). The efficient production of extracellular infectious virions requires the formation of the AC, and a disruption of AC morphogenesis leads to an increased production of noninfectious viral particles, as evidenced by increased particle/infectivity ratios (26, 28, 30). Although the formation of the AC has been shown to occur coincident with the expression of early viral proteins, the fully mature AC has been most well defined at late phases of infection and is maintained until lysis of the infected cell. To date, virus-encoded proteins that contribute to AC formation have been shown to include those that impact the cell cycle as well as virion proteins that are essential for virion assembly (31). In some cases, definition of the role of these viral proteins in AC formation has been confounded by their importance in virus replication and spread in HF cells. In this report, we have described the contribution of a nonessential envelope glycoprotein, gpUL132, encoded by UL132 to the formation of the AC and the contribution of gpUL132 to the early steps of infection. Our results indicate that gpUL132 is required for AC formation and the efficient production of infectious extracellular virus. Interestingly, the ΔUL132 virus failed to efficiently enter cells after virion attachment but did not alter the efficiency of cell-to-cell virus spread compared to WT virus-infected cells. Finally, we have demonstrated that only the cytosolic domain of gpUL132 expressed as a chimeric protein is required to rescue the *in vitro* phenotype of the WT virus. These results are consistent with previous findings that have demonstrated the importance of the AC for the optimal assembly of infectious extracellular virions.

## RESULTS

### Deletion of UL132 results in decreased infectious virus production but not viral genome replication or release of DNA-containing virion particles.

**FIG 1:**
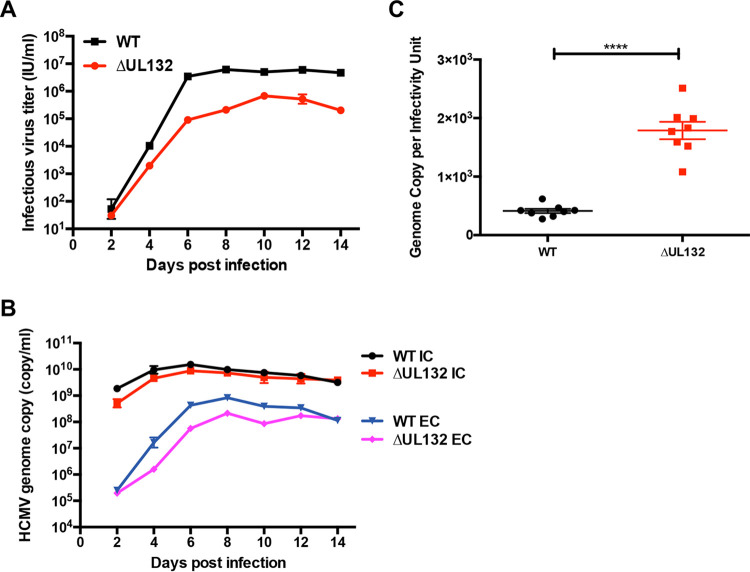
Decreased virus production from ΔUL132 HCMV-infected cells. (A) Infectious virus production from the extracellular supernatant. Confluent HFFs on 35-mm dishes were infected with either WT or ΔUL132 HCMV at an MOI of 0.1. At the indicated days postinfection (dpi), extracellular infectious virus was quantified as described in Materials and Methods. (B) Intracellular and extracellular HCMV genome copy numbers. Genome copy numbers were determined in the supernatants (extracellular [EC]) and from infected cells (intracellular [IC]) by qPCR of monolayers as described in Materials and Methods. Data are presented as the means and standard errors of the means (SEM) from three independent experiments. (C) HCMV particle-to-infectivity ratios. WT and ΔUL132 HCMV particle-to-infectivity ratios were calculated from extracellular virus at 7 dpi (*n* = 8); error bars denote SEM. Significances of differences between groups (*n* = 8 per group) were determined using the Student *t* test (****, *P* < 0.0001).

Consistent with previous findings, the deletion of UL132 resulted in the generation of a replication-competent virus (ΔUL132) that produced 1.5 to 2 logs fewer infectious extracellular virions than the WT parental virus (Fig. 1A) (13). In contrast to this difference in infectious virus production, the genome copy numbers in both WT and ΔUL132 virus-infected cells were nearly identical, and unexpectedly, the genome copy numbers were also nearly identical in cell-free particles released from WT- and ΔUL132-infected cells (Fig. 1B). These results indicated that neither a deficit in viral DNA replication nor the release of DNA-containing particles accounted for the decreased production of infectious virus by the ΔUL132 mutant virus. When the ratios of particles to infectious virus for WT and ΔUL132 extracellular viruses were compared, the ratio for the ΔUL132 virus was significantly higher than that for the WT virus, indicating that cells infected with ΔUL132 virus produced larger amounts of noninfectious particles (Fig. 1C).

**FIG 2:**
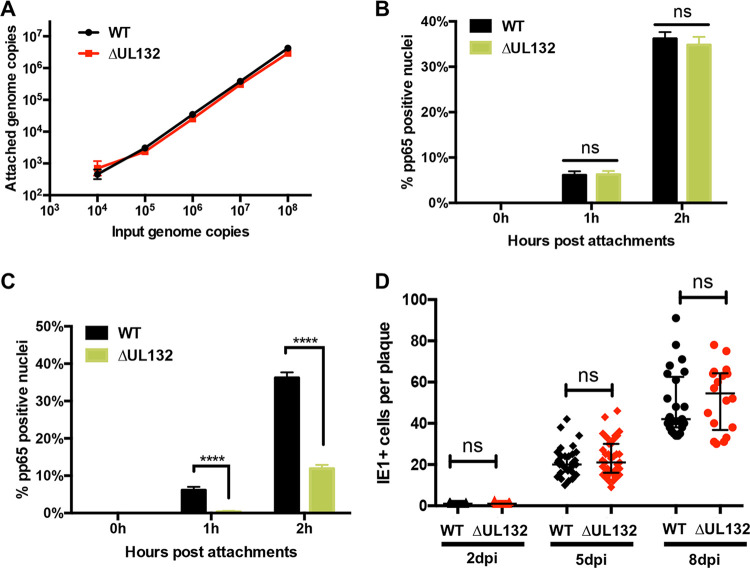
ΔUL132 HCMV attachment and cell-to-cell spread are similar to those of WT HCMV, but entry is delayed. (A) Attachments of ΔUL132 and WT viruses are similar. Genome copy numbers of attached HCMV virions are plotted over input genome copy numbers. Increasing numbers of genome copies of WT or ΔUL132 HCMV were added to confluent HFFs at 4°C and allowed to attach for 60 min. The cultures were then washed 4 times, and the remaining cell-associated HCMV genome copy numbers were quantified by qPCR. The data shown are from four independent experiments, and error bars denote SEM. (B and C) Entry of ΔUL132 is delayed compared to WT virus. Equal MOIs (5.5) (B) or equal genome copy numbers (10^8^) (B) of WT and ΔUL132 HCMV were added to HFFs and assayed for entry by detection of the tegument protein pp65 as described in Materials and Methods. Percentages of pp65-positive nuclei over the total nuclei per field were quantified. Eight random fields with ∼70 to 100 cells per field were counted and quantitated. Error bars denote SEM. Statistics were analyzed using the Student *t* test (****, *P* < 0.0001; ns, not significant). (D) ΔUL132 HCMV cell-to-cell spread is similar to that of the WT virus. Confluent HFFs were infected with WT or ΔUL132 HCMV at an MOI of 0.001 and overlaid with agarose. On the indicated day postinfection, the monolayers were fixed with paraformaldehyde, and the number of infected cells in individual plaques was determined by detection of the IE1 protein. IE1^+^ cells per plaque were quantitated and plotted at the indicated time points (*n* = ∼25 to 30); error bars denote medians with interquartile ranges. The experiments were repeated three times, and the data shown are from one experiment. Significance was determined using the Mann-Whitney test (ns, not significant).

As the product of UL132, gpUL132, is an abundant virion envelope protein, we next determined if the absence of this envelope protein impacted early steps in infectivity, including virion attachment and entry. Initially, we quantified the attachment of WT and ΔUL132 virions to HF cells using a binding assay carried out at 4°C to minimize entry. Comparable amounts of WT and ΔUL132 viruses attached to HF cells when the input genome copy numbers of the inocula were equivalent (Fig. 2A). To address whether gpUL132 could have a role in virus entry, HF cells were incubated with either equal genome copy numbers or equal multiplicities of infection (MOIs) of WT and ΔUL132 viruses, and virus entry was quantified by the detection of ppUL83 (pp65) in the nuclei of infected cells (32, 33). When inocula contained equal MOIs of WT and ΔUL132 viruses, pp65 could be detected in the nuclei of approximately 8% of WT- and ΔUL132-infected cells at 1 h (Fig. 2B; see also Fig. S2A in the supplemental material). At 2 h postinfection, nuclear pp65 was detected in approximately 38% of WT- and 37% of ΔUL132-infected cells (Fig. 2B; Fig. S2A). In contrast, when the inoculum for the entry assay contained equal genome copy numbers, we noted a marked difference in the entries of WT and ΔUL132 viruses as measured by pp65 nuclear translocation (Fig. 2C; Fig. S2B). After 1 h, pp65 was detected in the nuclei of about 8% of WT-infected cells but in none of the cells infected with ΔUL132 mutant virus (Fig. 2C; Fig. S2B). A similar difference was noted at 2 h postinfection, with WT virus infection resulting in nearly 40% of cells with nuclear pp65 compared to approximately 10% of cells infected with ΔUL132 (Fig. 2C; Fig. S2B). Together, these findings indicated that the defective phenotype of virions produced by cells infected with the ΔUL132 virus was not secondary to a defect in attachment but could be explained by a defect in entry by extracellular virions. Finally, to further investigate the role of gpUL132 in the early steps of infection, we compared the capacities of WT and ΔUL132 viruses to spread cell to cell by utilizing a plaque expansion assay. Cells were infected with a low MOI of either the WT or ΔUL132, and plaque expansion was quantified by the number of immediate early 1 (IE1) protein-expressing cells per plaque as a function of time postinfection. Interestingly, the rates of plaque expansion were similar for both WT and ΔUL132 virus-infected cells, indicating that the deficit in cell entry by ΔUL132 extracellular virus did not extend to cell-to-cell virus spread as measured in this assay (Fig. 2D; Fig. S2C). This experiment was also performed with equal genome copy number inocula for WT and ΔUL132 viruses, and no differences in cell-to-cell spread were observed (Fig. S2D). Finally, to relate the detection of IE1-expressing cells to plaque size, we quantified the areas of plaques from the plaque expansion assay and noted similar physical sizes of plaques as defined by the total number of cells in the involved area of the monolayer (Fig. S2E).

### Extracellular viral particles produced in ΔUL132 virus-infected cells are stable but differ in protein composition compared to WT virus.

A potential explanation for the decreased infectivity and increased particle-to-infectious virus ratio of extracellular ΔUL132 virions was that gpUL132 played an important role in virion stability such that extracellular virions lacking gpUL132 were labile and infectivity decreased during assays of infectivity and entry. To rule out this possibility, we compared the maintenance of input infectivity of extracellular WT and ΔUL132 virions by quantifying residual infectivity following incubations at room temperature, 37°C, and after freezing at −20°C. There were no significant differences in the stability of infectivity between the WT and ΔUL132 viruses under these conditions, indicating that decreased *in vitro* stability of infectious virions did not account for the phenotype of the ΔUL132 virus (Fig. S1).

10.1128/mBio.02044-20.1 FIG S1 ΔUL132 HCMV stability. Totals of 2 × 10^3^ IU/ml (A) or 4 × 10^4^ IU/ml (B and C) WT and ΔUL132 HCMV were incubated at room temperature (*n* = 10) (A) or 37°C (*n* = 6) (B) for the indicated times and then titrated using indirect immunofluorescence with an antibody against the IE1 protein. Data were normalized to the virus infectivity units at 0 h. Error bars denote SEM. (C) WT and ΔUL132 HCMV were kept frozen at −20°C and thawed every 12 h. At each thaw, 100 μl of viruses was taken for titration. Data from 5 independent experiments were normalized and averaged. Statistics were analyzed using two-way analysis of variance (ANOVA) with Tukey’s multiple-comparison test (****, *P* < 0.0001; ***, *P* < 0.001; *, *P* < 0.05; ns, not significant). Error bars denote SEM. Download FIG S1, DOCX file, 0.1 MB. Copyright © 2020 Wu et al. 2020 Wu et al. This content is distributed under the terms of the Creative Commons Attribution 4.0 International license.

10.1128/mBio.02044-20.2 FIG S2 ΔUL132 HCMV entry is delayed but is not defective in cell-to-cell spread. (A and B) For quantitation of entry, equal MOIs (5.5) (A) or equal genome copy numbers (10^8^) (B) of WT and ΔUL132 HCMV were added to HFFs and processed for detection of the tegument protein pp65 (red) or the immediate early 1 protein (IE1) (green) by immunofluorescence and confocal microscopy as described in Materials and Methods. DAPI staining was used to identify the nucleus (blue). Results are shown for time points of 0 h, 1 h, and 2 h postattachment. Bar, 50 nm. (C) ΔUL132 HCMV cell-to-cell spread is not delayed. Confluent HFFs were infected with WT or ΔUL132 HCMV at an MOI of 0.001 and then overlaid with agarose. On the indicated day postinfection, the monolayers were fixed with paraformaldehyde, and the number of infected cells in individual plaques was determined by immunofluorescence detection of the IE1 protein. Representative images of HCMV plaques as detected are shown. The experiments were repeated three times, and the data shown are from one experiment. (D) Results when inocula were of equal genome copy numbers to demonstrate equal cell-to-cell spread of WT and ΔUL132 viruses irrespective of the input inoculum. (E) Areas of plaques from cell-to-cell spread demonstrating similar-size plaques for both WT and ΔUL132 viruses. Plaque areas were quantified by measurement of DAPI-stained cells, and median areas were compared by Mann-Whitney tests (NS, not significant). Note the similar distributions of plaque sizes in monolayers infected by either virus. Download FIG S2, DOCX file, 0.3 MB. Copyright © 2020 Wu et al. 2020 Wu et al. This content is distributed under the terms of the Creative Commons Attribution 4.0 International license.

**FIG 3:**
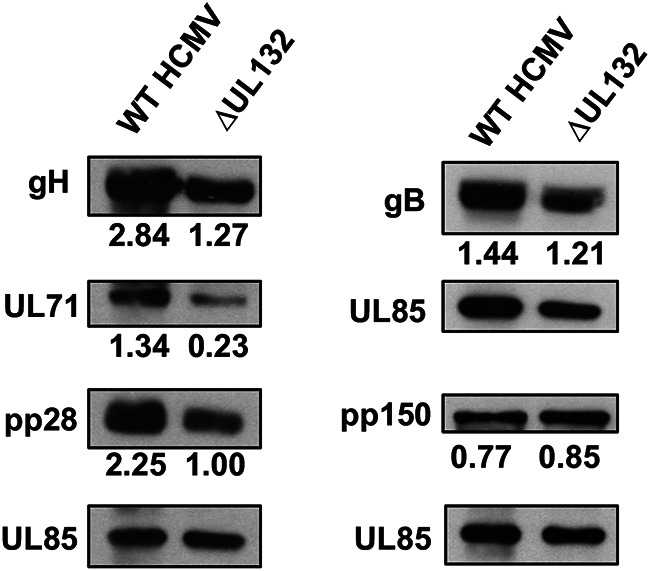
Deletion of UL132 alters the viral protein composition of extracellular virions. Confluent HFFs were infected with WT or ΔUL132 HCMV at an MOI of 0.05, and extracellular virions were pelleted on day 10 postinfection by high-speed centrifugation through a 25% sorbitol cushion and analyzed by immunoblotting. Equal numbers of genome copies of virions were loaded per lane. Panels show representative blots of envelope proteins (gB and gH), tegument proteins (UL71, pp28, and pp150), and minor capsid protein (UL85) in extracellular particles collected from infected cells. The relative signals from the viral protein normalized to the signal from UL85 are indicated. The experiments were repeated twice, and the data shown are from one experiment.

We then surveyed the relative abundances of several essential virion proteins in WT and ΔUL132 extracellular virions by immunoblotting. Compared to WT virions, ΔUL132 virions contained small amounts of gH and the outer tegument proteins ppUL71 and pp28 when normalized to the amount of the capsid protein pUL85 (Fig. 3). Similarly, UL132 extracellular virions contained smaller amounts of gB. In contrast, WT and UL132 virions contained equivalent amounts of the inner tegument protein pp150 (Fig. 3). Because approximately equal genome copy numbers of extracellular particles from WT and ΔUL132 virus-infected cells were analyzed in these experiments, these results argued that as a population, extracellular particles produced by ΔUL132 mutant virus-infected cells contained decreased amounts of envelope and outer tegument proteins (34, 35). This finding raised the possibility that the deletion of UL132 leads to the assembly of a heterogeneous population of extracellular virions, as illustrated by the altered composition of the envelope and tegument proteins. The heterogeneity in extracellular virions could then lead to a decrease in infectivity of the population of ΔUL132 virus, a high particle-to-infectivity ratio, and an entry deficit when equivalent numbers of ΔUL132 and WT particles were compared (Fig. 2C). Thus, these findings argued that the phenotype of the ΔUL132 virus could be explained by the role of gpUL132 in the assembly of infectious progeny and not by its function(s) in the envelope of extracellular virions.

### The morphogenesis of the virus assembly compartment is altered in ΔUL132 HCMV-infected cells.

**FIG 4:**
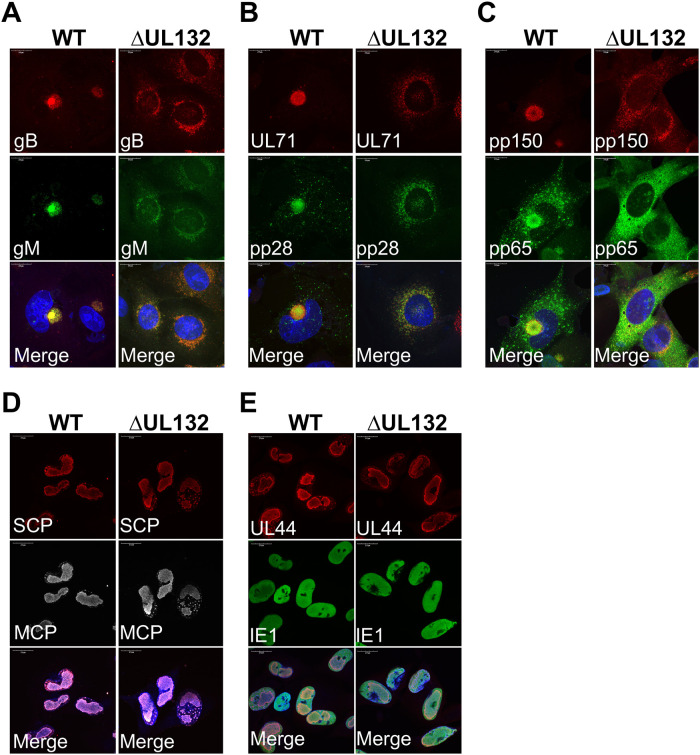
Viral proteins fail to accumulate in the juxtanuclear assembly compartment (AC) during ΔUL132 HCMV infection. Confluent HFFs were infected with WT or ΔUL132 HCMV at an MOI of 3. Infected cells were fixed with paraformaldehyde on day 4 postinfection, processed for immunofluorescence, and analyzed by confocal microscopy. Representative images of HCMV envelope proteins gM and gB (A); tegument proteins UL71, pp28, pp150, and pp65 (B and C); major capsid protein (MCP) and smallest capsid protein (SCP) (D); and nonstructural proteins UL44 and IE1 (E) are shown. All antibodies used were mouse mAbs. Magnification, ×120. The experiments were repeated three times, and the data shown are from one experiment.

In previous studies, we and others have demonstrated the importance of the virus-induced reorganization of intracellular membranes and the resulting formation of the juxtanuclear virus assembly compartment (AC) for the efficient production of infectious virions (15–18). More recently, we have shown that a failure to form the AC through either deletion of vmiRNAs or inhibition of the virus-induced disruption of the Golgi apparatus resulted in decreased production of infectious virions and the production of noninfectious extracellular virus populations characterized by high particle/infectious virus ratios (26, 30). These previous findings suggested a potential role of UL132 in the morphogenesis of the AC and the subcellular location of viral tegument and envelope proteins (Fig. 4). The HCMV envelope proteins gB and gM and the tegument proteins pp28, pp65, pp150, and UL71 accumulated in the juxtanuclear AC in WT virus-infected cells yet failed to localize in a similar juxtanuclear site in ΔUL132 virus-infected cells (Fig. 4A to C). In contrast to these findings, the nuclear localizations of the nonstructural proteins IE1 and ppUL44, the major capsid protein (MCP) UL86, and the smallest capsid protein (SCP) UL48.5 were similar in both WT and ΔUL132 HCMV-infected HF cells (Fig. 4D and E). Thus, the deletion of UL132 leads to a mislocalization of viral proteins that accumulate in the cytoplasm of virus-infected cells.

**FIG 5:**
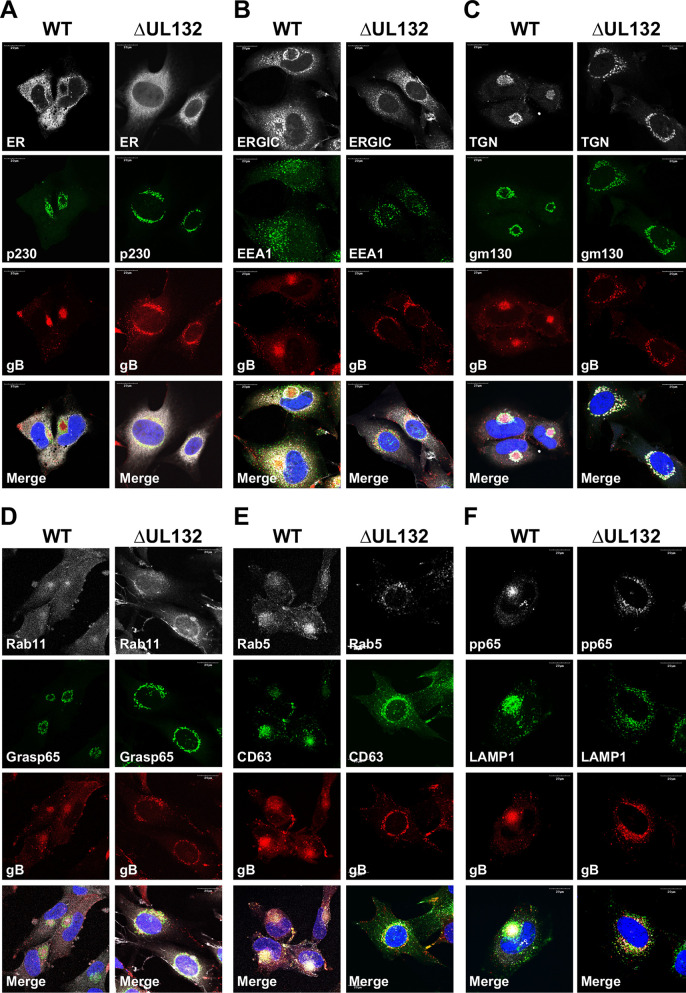
Membranous compartments of the secretory pathway do not reorganize during ΔUL132 HCMV infection. HFFs infected with WT or ΔUL132 HCMV were fixed and analyzed as described in the legend of Fig. 4. Infected HFFs were stained with viral proteins gB and pp65 to identify infected cells. Representative images of intracellular compartments, including the endoplasmic reticulum (ER), the ER-Golgi intermediate compartment (ERGIC), the *trans*-Golgi network (TGN) (p230), *cis*-Golgi (gm130 and Grasp65), endosomal systems (EEA1, Rab5, and Rab11), late endosomes (CD63), and lysosomes (LAMP1) are shown. Magnification, ×120. The experiments were repeated three times, and the data shown are from one experiment.

**FIG 6:**
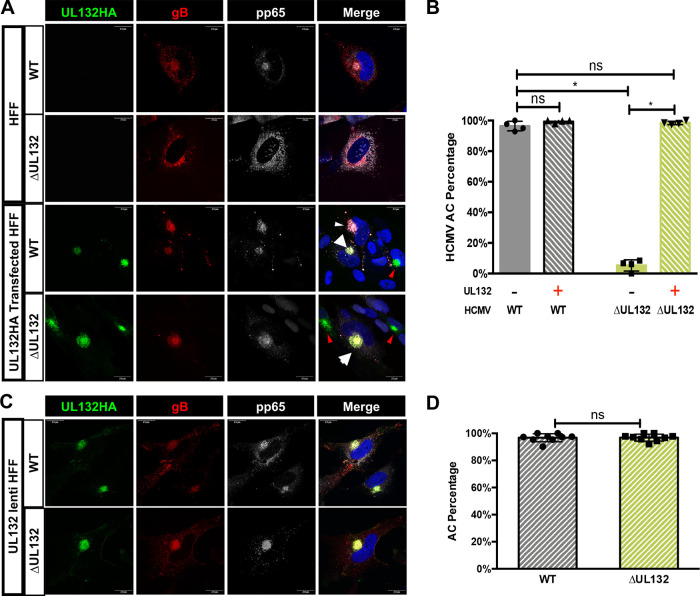
In *trans* expression of gpUL132 rescues the assembly compartment formation phenotype in ΔUL132 HCMV-infected cells. (A and B) Transient expression of gpUL132 rescues the AC formation phenotype in ΔUL132 HCMV-infected cells. Confluent HFFs were mock treated or electroporated with a vector expressing UL132 and then infected with WT or ΔUL132 HCMV. Infected cells were harvested at 4 dpi, fixed, processed for immunofluorescence staining of gB and pp65, and analyzed using confocal microscopy. (A) Transient expression of gpUL132 rescues the AC in ΔUL132 HCMV-infected cells. Representative images are shown. The images in the top two rows illustrate the morphology of the AC as demarcated by gB and pp65 staining in WT and ΔUL132 HCMV-infected cells compared to the cells electroporated with a vector expressing UL132 and then infected with the respective viruses in the bottom panels. Magnification, ×120. (B) Quantification of AC formation in transiently expressed gpUL132-infected HFFs. (C and D) The WT AC phenotype is rescued in ΔUL132 HCMV-infected UL132-expressing cells. Confluent UL132-expressing HFFs were infected with WT or ΔUL132 HCMV. Infected cells were harvested at 4 dpi; fixed; processed for immunofluorescence detection of gB, pp65, and HA-tagged UL132; and analyzed using confocal microscopy. (C) Representative images of AC formation in UL132-expressing cells. Magnification, ×120. (D) Quantification of AC formation in UL132-expressing HFFs during infection. ACs that formed in nine random fields with 35 to 45 cells per field were counted, and the percentage of ACs per field was plotted. A total of 349 WT HCMV-infected cells and 320 ΔUL132 HCMV-infected cells were counted. Error bars denote standard deviations (SD) of the means. Significance was determined using the Student *t* test (*, *P* < 0.05; ns, not significant). The experiments were repeated twice, and the data shown are from one experiment.

The altered intracellular localization of the virion structural proteins in ΔUL132 virus-infected cells could be explained as a direct effect on either viral protein trafficking or an aberrant cellular response to HCMV infection that is required for membrane reorganization and accumulation of virion structural proteins in the AC. To determine if UL132 had a role in the reorganization of intracellular membranes observed during AC morphogenesis, we compared the localizations of protein components of the cellular secretory system in WT and ΔUL132 virus-infected cells. Consistent with previous studies, the resident endoplasmic reticulum protein calreticulin did not overlap viral proteins that accumulated in the AC of WT virus-infected cells (Fig. 5A) (15–17). In contrast, the localization of the endoplasmic reticulum-Golgi intermediate compartment (ERGIC) protein ERGIC53 partially overlapped that of viral proteins accumulating in the AC of WT virus-infected cells, whereas this protein failed to colocalize with viral proteins in ΔUL132 virus-infected cells (Fig. 5B). The *cis*-Golgi proteins gm130 and Grasp65 and the *trans*-Golgi proteins TGN46 and p230 appeared to surround viral proteins that accumulated within the AC in WT virus-infected cells (Fig. 5A, C, and D). In contrast, in ΔUL132 virus-infected cells, membranes identified by these cellular proteins were dispersed and did not localize to the juxtanuclear site of the AC in WT virus-infected cells (Fig. 5A, C, and D). The reorganization of the endosomal system that has been well described in WT virus-infected cells was not observed in ΔUL132 virus-infected cells as shown by the distribution of the early endosomal proteins EEA1, Rab5, and Rab11 as a marker for the recycling endosomal compartment (Fig. 5B, D, and E). Finally, the reorganization of the late endosomal compartment in WT virus-infected cells that could be identified by the expression of CD63 and LAMP1 surrounding the AC was noticeably altered in ΔUL132-infected cells (Fig. 5E and F). We quantified the number of cells with a morphologically recognizable AC defined by the presence of a compact juxtanuclear structure containing virion structural proteins, specifically envelope glycoproteins and the tegument protein pp65, that were wrapped in host cell proteins of the secretory pathway. Using these criteria, there was a near absence of infected cells with a mature AC in ΔUL132 virus-infected cells (<5%) compared to WT virus-infected cells (96%) (Fig. 6A and B). Finally, the defect in AC formation in ΔUL132 virus-infected cells was not the result of a delay in the kinetics of AC formation in ΔUL132-infected cells, as a mature AC was not detected in ΔUL132-infected cells even after a prolonged period of infection (6 days) (Fig. S3). Together, these findings indicated that in the absence of a single virion envelope protein, gpUL132, virus-infected cells failed to undergo the stereotypic membrane reorganization leading to AC morphogenesis that is characteristic of HCMV infection in HF cells. Interestingly, progeny virion populations with a high particle-to-infectivity ratio have also been described previously in infected cells that failed to form recognizable ACs (26, 30).

10.1128/mBio.02044-20.3 FIG S3 AC formation kinetics in HCMV infection. Confluent HFFs were infected with WT or ΔUL132 HCMV at an MOI of 2. Infected cells were collected at the indicated days postinfection, fixed with paraformaldehyde, processed for immunofluorescence staining (green, TGN; red, pp65; gray, gB; blue, nucleus), and analyzed using confocal microscopy. All antibodies used were mouse monoclonal antibodies. Representative AC morphologies at different time points are shown. Magnification, ×100. The experiments were repeated twice, and the data shown are from one experiment. Download FIG S3, DOCX file, 0.1 MB. Copyright © 2020 Wu et al. 2020 Wu et al. This content is distributed under the terms of the Creative Commons Attribution 4.0 International license.

### In *trans* expression of gpUL132 rescues assembly compartment defects in ΔUL132 HCMV-infected human foreskin fibroblasts (HFFs).

To formally demonstrate that the defects in the morphogenesis of the AC, including both cellular membrane reorganization and virion protein accumulation, in ΔUL132 HCMV-infected cells were secondary to the deletion of UL132 and not secondary to undefined viral functions in this region of the viral genome, we transiently expressed a hemagglutinin (HA)-tagged gpUL132 (UL132HA) in HF cells, followed by infection with WT or ΔUL132 HCMV. We noted that in *trans* expression of UL132 rescued the defects in assembly compartment formation in ΔUL132 virus-infected cells and restored the number of cells with mature assembly compartments to a value (98%) similar to that in WT virus-infected cells (Fig. 6A and B). The complementation of deficits in the ΔUL132 virus was further demonstrated in assays using a lentivirus-transduced HF cell line that stably expressed gpUL132 (Fig. 6C and D). Together, these data strongly argued that viral UL132 was required for host cell membrane reorganization leading to AC formation and the accumulation of virion structural proteins in this previously described cytoplasmic site of virus assembly.

### The cytosolic domain of gpUL132 is adequate to rescue assembly compartment formation and the production of infectious virus in ΔUL132-infected HF cells.

**FIG 7:**
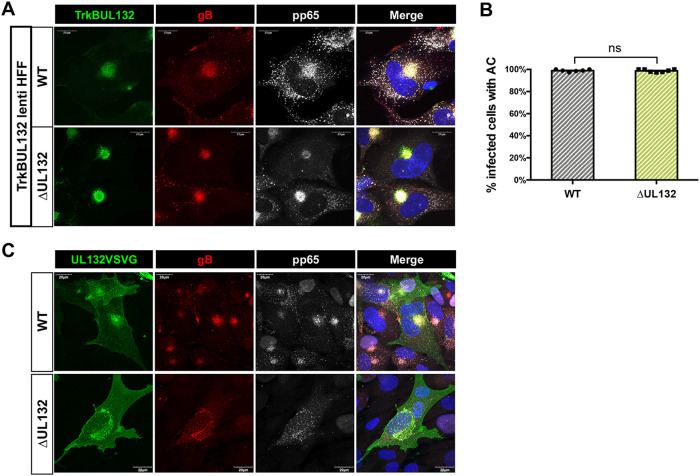
The cytoplasmic domain of UL132 is sufficient to rescue AC formation during ΔUL132 HCMV infection. (A and B) TrkBUL132-expressing cells rescued AC formation following ΔUL132 virus infection. Confluent TrkBUL132 (Myc-tagged)-expressing cells were infected with WT or ΔUL132 HCMV at an MOI of 3. At 4 dpi, infected cells were harvested and processed for immunofluorescence to detect the expression of the Myc, gB, or pp65. (A) TrkBUL132-expressing cells rescued AC formation following ΔUL132 HCMV infection. Representative images are shown (magnification, ×120). (B) Quantification of AC formation percentages in HCMV-infected TrkBUL132-expressing HFF cells. Random fields with 70 to 95 cells per field were counted and quantified. Error bars denote SEM. Significance was determined using the Student *t* test (ns, not significant). The experiments were repeated twice, and the data shown are from one experiment. (C) Transiently expressed UL132VSVG cannot rescue AC formation in ΔUL132 HCMV-infected HFFs. A UL132VSVG (HA tag)-expressing vector was electroporated into HFFs, and HFFs were then infected with WT or ΔUL132 HCMV at an MOI of 2. Coverslips were collected, fixed, and analyzed using immunofluorescence and confocal microscopy. Representative images from two independent experiments are shown.

gpUL132 is predicted to be a type I transmembrane protein consisting of an extracellular domain (ectodomain), a transmembrane domain, and a cytosolic domain (13). To identify the gpUL132 domain responsible for the formation of the AC and the efficient production of extracellular infectious virus, we utilized a previously described chimeric fusion protein, TrkBUL132, with the ectodomain and transmembrane domain from a rat receptor tyrosine kinase, TrkB, fused to the cytosolic domain of gpUL132 (14). We have shown that this chimeric protein can be incorporated into virions produced by ΔUL132 virus-infected cells, indicating that it localized to sites of virus assembly in HCMV-infected cells (14). The morphogenesis of the mature AC could be demonstrated in the ΔUL132-infected TrkBUL132-expressing HF cell line (Fig. 7A). When quantified, ΔUL132 virus infection of TrkBUL132-expressing HF cells resulted in numbers of cells with an AC comparable to those in WT virus-infected cells (Fig. 7B). To establish that the expression of the ectodomain and transmembrane domain of gpUL132 was not required for the morphogenesis of the AC, we transiently expressed the ectodomain and transmembrane domain of UL132 fused with the vesicular stomatitis virus (VSV) G protein cytosolic domain. The expression of the chimeric gpUL132:VSV G fusion protein in *trans* failed to complement the formation of the AC in ΔUL132 virus-infected cells (Fig. 7C). These results argued that the cytosolic domain of gpUL132 was sufficient to rescue the formation of the AC during infection with the ΔUL132 virus.

**FIG 8:**
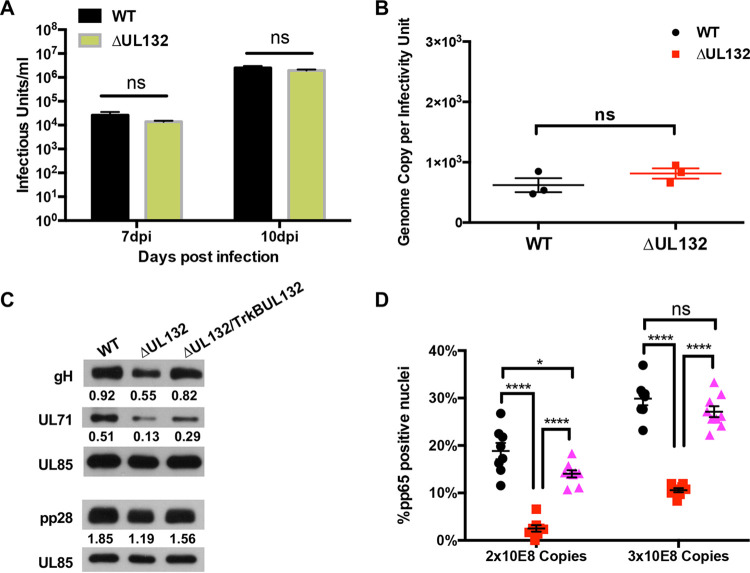
The cytoplasmic domain of UL132 is sufficient to rescue virus production and entry in ΔUL132-infected TrkBUL132-expressing cells. (A and B) Confluent TrkBUL132-expressing cells were infected with WT or ΔUL132 HCMV at an MOI of 0.1. Supernatants were collected at the indicated days postinfection. Extracellular infectious virus was titrated, and qPCR was performed to quantify the extracellular HCMV genome copy numbers as described in the legend of Fig. 1. Data are the means and SD from three independent experiments. (A) HCMV production. (B) HCMV progeny particle-to-infectious unit ratios. Significance was determined using the Student *t* test (ns, not significant). (C) Progeny of ΔUL132 virions from TrkBUL132-expressing cells contain levels of envelope and outer tegument proteins similar to those in WT virions. Extracellular virions from WT and ΔUL132-infected HF cells and from ΔUL132-infected TrkBUL132-expressing cells were collected by centrifugation. Equal numbers of genome copies of each virus preparation were analyzed by immunoblotting to quantify virion protein expression. Amounts of gH, ppUL71, and p28 were normalized to the amount of UL85. (D) Progeny virions from ΔUL132-infected TrkBUL132 HFFs are not defective in entry. Equal numbers of genome copies of cell-free virus from WT (black circles)- or ΔUL132 (red squares)-infected HFF cells or progeny from ΔUL132-infected TrkBUL132 HFFs (magenta triangles) were added to HFFs and processed for entry assays as described in Materials and Methods. Results are shown for time points of 2 h postattachment. Percentages of pp65-positive nuclei over the total nuclei per field were quantified. Eight random fields with ∼70 to 100 cells per field were counted and quantitated. Error bars denote SEM. Statistics were analyzed using the Student *t* test (****, *P* < 0.0001; *, *P* < 0.05; ns, not significant).

To determine if the function of the cytosolic domain of gpUL132 expressed by the chimeric protein TrkBUL132 was sufficient to complement the production of infectious virions in ΔUL132 virus-infected cells, we quantified infectious virus production following ΔUL132 or WT virus infection of the stable HF cell line expressing the chimeric TrkBUL132 protein. Virus yields were similar in the supernatants from infected TrkBUL132-expressing cells regardless of whether these cells were infected with the WT or ΔUL132 virus, indicating that the cytosolic domain of gpUL132 was sufficient to support WT levels of virus replication (Fig. 8A). More importantly, the particle-to-infectivity ratio of extracellular virus released from TrkBUL132-expressing cells infected with ΔUL132 was similar to that of extracellular virus produced by WT-infected TrkBUL132 cells (Fig. 8B). Furthermore, the abundances of virion envelope and outer tegument proteins (gH, pp28, and ppUL71) in extracellular particles from ΔUL132 and WT virus-infected TrkBUL132-expressing cells were comparable, suggesting that the formation of the AC was linked to the abundance of envelope and outer tegument proteins in extracellular virions (Fig. 8C). Finally, we compared the entries of populations of extracellular particles produced by WT- and ΔUL132-infected TrkBUL132 cells to determine if virions derived from ΔUL132-infected TrkBUL132 cells could efficiently enter cells. Complementation of the deletion of UL132 resulted in the production of extracellular particles in ΔUL132-infected TrkBUL132 cells that entered cells as efficiently as the WT virus (Fig. 8D). In summary, these results demonstrated that the cytosolic domain of gpUL132 provided an essential function(s) required for the morphogenesis of the AC and the assembly of extracellular virions that were phenotypically similar, in terms of their content of envelope and outer tegument proteins and their capacity to efficiently enter HF cells, to virions that were produced by WT-infected cells.

## DISCUSSION

In this report, we have described a previously unknown function of an abundant HCMV virion envelope glycoprotein, gpUL132, in the production of infectious extracellular virions. Consistent with previous findings, the deletion of the UL132 reading frame resulted in a 1- to 2-log decrease in virus yield compared to the WT parental virus; however, a more striking phenotype of this mutant virus was the contribution of UL132 to the morphogenesis of the AC. The deletion of UL132 resulted in altered AC morphogenesis, including viral protein trafficking to this well-described cytoplasmic site of virus assembly. Similarly, intracellular membranes, including the Golgi apparatus and components of the endocytic pathway that are extensively reorganized in WT virus-infected cells and localized to the juxtanuclear AC, were scattered throughout the cytoplasm in cells infected with the ΔUL132 virus. In addition, localization of the MTOC to the juxtanuclear site, a characteristic of the virus-induced AC, was not observed in ΔUL132-infected cells (15, 21). The transient or constitutive expression of UL132 in the absence of virus infection failed to induce membrane reorganization, indicating that gpUL132 must function in concert with other viral functions to promote AC morphogenesis. In contrast, the transient expression of UL132 in cells infected with the ΔUL132 mutant virus exhibited membrane reorganization and viral protein trafficking to the AC that were indistinguishable from those of WT virus-infected cells, indicating the requirement of UL132 expression for AC formation. In addition, the rescue of the ΔUL132 virus by infection of UL132-expressing cells resulted in the normalization of infectious virus production in multistep virus yield assays. Thus, our findings provided additional evidence for a direct linkage between AC formation and the efficient assembly of infectious virions, a correlation noted in previous reports (26, 30). However, in contrast to previous reports that have described the contribution of cellular proteins to AC formation, such as those targeted by vmiRNAs or inhibited by agents targeting key SNARE proteins such as Syntaxin 5 or the Golgi-resident protein Grasp65, we have provided evidence that a single virion protein provides an essential function required for the morphogenesis of the virus-induced AC (24–30).

The product of UL132, gpUL132, is a type I membrane protein that is relatively abundant in the virion envelope (11). Although the deletion of UL132 resulted in a decreased yield of infectious virus, to date, gpUL132 has not been shown to have a role in the early steps of virus infection. It was therefore of interest that the mutant ΔUL132 virus appeared to have a deficit in entry but not attachment or cell-to-cell spread, suggesting a potential role of gpUL132 in early events of infection. However, this deficit in entry was observed only when equal numbers of genome copies of ΔUL132 and WT viruses were compared and not when equal MOIs of inocula of ΔUL132 and WT viruses were assayed. Thus, virions lacking gpUL132 do not appear to have an absolute defect in entry. These findings suggested either that a minor population of ΔUL132 virions present in the heterogeneous population of virions produced by ΔUL132-infected cells was fully competent in the entry assay or that the entry function(s) provided by gpUL132 could be replaced by those of other virion proteins when infections were carried out with a larger number of particles. Although we cannot formally exclude the latter possibility, increasing the inoculum size of virions produced by ΔUL132-infected cells could also have increased minor populations of virions that expressed sufficient amounts of essential envelope and tegument proteins to allow efficient virus entry. Such a mechanism was consistent with the heterogeneity of extracellular virions from ΔUL132-infected cells that were characterized by a high particle/infectivity ratio and an altered composition of virion envelope and tegument proteins. Finally, perhaps the most compelling evidence supporting this mechanism was the phenotype of ΔUL132 virions produced in TrkBUL132-expressing cells. Complemented virions entered cells as efficiently as WT virions, exhibited near-WT particle/infectious virus ratios, and contained amounts of outer tegument and envelope proteins similar to those of WT virions. Of note, virions produced by ΔUL132-infected TrkBUL132 cells contained only the cytosolic domains of gpUL132 and not the ectodomain and transmembrane domain of this type I membrane glycoprotein. From these data, we argue that UL132 contributes an essential function required for the morphogenesis of the AC in infected cells and that the formation of the AC plays a major role in the efficient assembly of infectious extracellular virions.

Although the AC induced during productive HCMV infection has been the subject of extensive study, a unifying mechanism describing its function in virus assembly has not been presented. The localization and concentration of virus-encoded proteins that are incorporated into the maturing virion during the cytoplasmic phase of virus assembly to the AC, including envelope and outer tegument proteins, have been postulated to be major functions of the AC (15). Other investigators have suggested that virion components are acquired sequentially as the partially tegumented particles pass through the concentrically organized membranes of the AC (18). These and other proposed roles for the AC in virus assembly share a step that includes the coalescence of membranous compartments of the infected cell at a single location, thereby increasing the likelihood that virions containing a full ensemble of proteins, both virus encoded and host derived, will be assembled. Thus, the AC could function to increase the probability that infectious virion progeny, and not defective particles, will be assembled. This postulated mechanism would argue that in the absence of a fully formed AC, the likelihood of the assembly of infectious virions will decrease, and a corresponding increase in the assembly of noninfectious particles would be observed. A corollary of this mechanism would be that the heterogeneity in virion protein content would be most apparent for viral proteins that are believed to be acquired during the cytoplasmic phase of assembly, particularly those that are thought to be membrane associated and not those of the inner tegument, such as pp150. Consistent with this mechanism was the observation that the content of the envelope glycoprotein gH was shown to be significantly reduced in virions from ΔUL132-infected cells. Similarly, there was less gB present in ΔUL132 than in WT virions, albeit this was a less significant difference than what was observed with gH. It is important to note that neither gB nor gH is required for the production of particles, as mutant viruses with a deletion of either gH or gB can produce extracellular, albeit noninfectious, particles (2, 36). We also noted that the levels of two well-described outer tegument proteins that have been shown to be membrane associated, pp28 and ppUL71, were decreased in extracellular virions recovered from ΔUL132-infected cells compared to virions from WT-infected cells. In support of this mechanism for the AC in virus assembly, when the morphogenesis of the AC was complemented in ΔUL132 virus-infected UL132-expressing cells, we noted a normalization of the quantities of virion gH, pp28, and ppUL71 compared to their expression in virions from WT virus-infected cells.

In summary, the findings presented in this report together with those from previous studies strongly argue that the formation of the AC in the infected cell is required for the optimal production of infectious extracellular virions. Although a unifying mechanism to account for the role of the AC in the production of infectious virus remains to be described, an increased particle-to-infectious virus ratio and a decreased content of virion envelope and outer tegument proteins in extracellular virions produced by infected cells with altered ACs suggest that later stages of cytoplasmic virion assembly are facilitated by the formation of the AC. The critical role of the cytosolic domains of UL132 in the formation of the AC in the infected cell was not observed when UL132 was expressed in noninfected cells. This observation strongly suggested that this viral protein interacts with other viral and cellular functions, which in turn leads to the reorganization and relocalization of cellular membranes to form the AC. The identification of the components of these interactions will likely provide important new insight into the virus-induced reorganization of intracellular membranes that is characteristic of the HCMV-infected cell.

## MATERIALS AND METHODS

### Antibodies, cells, and viruses.

The mouse monoclonal hybridoma antibodies (mAbs) against viral proteins utilized in this project included antibodies to gB (27-156), gM (IMP), pp65 (28-19), pp150 (XPA36-14), pp28 (41-18), UL71 (7D9-2), MCP (28-4), SCP (11-2-23), UL44 (28-21), and IE1 (p63-27) and anti-CD63 mouse mAb (11, 15, 16, 20, 29, 30). The commercially available antibodies used in these studies were anti-gm130 mAb (BD Transduction Laboratories, San Jose, CA), anti-p230 mouse mAb (BD Transduction Laboratories), anti-TGN46 sheep polyclonal antibody (pAb; SeroTec), anti-ERGIC53 pAb (Sigma-Aldrich, St. Louis, MO), anti-calreticulin rabbit pAb (Affinity Bioreagents, Golden, CO), anti-EEA1 mouse mAb (BD Transduction Laboratories), anti-Rab11 rabbit mAb (Cell Signaling, Danvers, MA), anti-Rab5 mouse mAb (BD Transduction Laboratories), anti-Grasp65 mouse mAb (Novus Biologicals), anti-LAMP1 mouse mAb (Iowa Developmental Studies Hybridoma Bank), anti-HA epitope mouse mAb (Covance, Madison, WI), and anti-Myc epitope mouse mAb (Cell Signaling). Human cytomegalovirus strain AD169 and the previously described ΔUL132 HCMV strain were propagated in human foreskin fibroblast (HFF) cells grown in Dulbecco’s modified Eagle medium (DMEM) (Corning Cellgro, Manassas, VA) supplemented with glucose, glutamine, sodium pyruvate, fetal bovine serum (5%), penicillin (100 U/ml), and streptomycin (100 μg/ml) (13). Virus stocks were prepared by collecting supernatants and cells from cultures exhibiting 100% cytopathic effect, clarified to remove cells and debris, and stored at −80°C until utilized (26). 293T cells were cultured in DMEM supplemented with 10% fetal bovine serum, penicillin, and streptomycin. Lentiviruses were constructed in 293T cells using the Lenti-X packaging system (TaKaRa, Mountain View, CA, USA), except that Polyfect transfection reagent (Qiagen, Germantown, MD) was used as a transduction reagent. UL132- and TrkBUL132-expressing lenti HFF stable cell lines were constructed using lentiviral transduction of HFFs under puromycin selection and passaged in DMEM supplemented with glucose, glutamine, sodium pyruvate, 5% fetal bovine serum, penicillin-streptomycin, and puromycin.

### Plasmids and constructs.

pcUL132 and pcDNA-TrkBUL132 were previously described (14). The chimeric protein UL132VSVG containing the UL132 ectodomain with an HA tag and the vesicular stomatitis virus G protein (VSV G) transmembrane and cytoplasmic domains was generated using overlapping PCR with the primer pair 5′-GATATCAAGCTTGCCGCCACCATGCCGGCCCCGCGGGGTCTCCTTC-3′ and 5′-GATTAACCCTATGATAAAGAAAAAAGCCAGCACTTTCATGATTTC-3′ and the primer pair 5′-CGCAATGACGAAATCATGAAAGTGCTGGCTTTTTTCTTTATCATAGGGTTAATC-3′ and 5′-ACTAGTCTCGAGTTACTTTCCAAGTCGGTTCATCTC-3′ and cloned into the pcDNA3.1 construct using the HindIII and XhoI restriction enzyme sites.

### Virus infectivity and qPCR.

To determine viral titers, fibroblasts were infected with serial dilutions of supernatants and incubated for 24 h. After fixation in ethanol, cells were stained with an antibody directed against IE1 (p63-27) followed by fluorescein isothiocyanate (FITC)-labeled goat anti-mouse IgG. The number of IE1-positive (IE1^+^) cells was counted, using a fluorescence microscope, at the lowest dilution that gave at least 100 IE1^+^ cells/well to determine the number of infectious units (IU) per milliliter (26). The genome copy number was determined by quantitative PCR (qPCR) using primers 5′-AGGTCTTCAAGGAACTCAGCAAGA-3′ and 5′-CGGCAATCGGTTTGTTGTAAA-3′ with the probe 5′-56-FAM-AACCCGTCAGCCATTCTCTCGGC-36-TAMSp-3′ (FAM/TAMRA) and normalized to WHO international standards to provide absolute quantification.

### Confocal microscopy.

HFFs were grown on glass coverslips within 24-well plates and fixed in 4% paraformaldehyde (PFA) in phosphate-buffered saline (PBS) (37). Cells were permeabilized with 0.1% Triton X-100 in PBS prior to blocking with 5% normal goat serum in PBS. Coverslips were then incubated with monoclonal antibodies or polyclonal primary antibodies in 5% normal goat serum in PBS with 0.2% Tween 20 (PBST). When using rabbit polyclonal antibodies, the ChromPure human IgG Fc fragment (Jackson ImmunoResearch, West Grove, PA) was included for all primary and secondary antibody incubations. After incubation with primary antibodies, coverslips were washed in PBS containing 0.1% Tween 20. Alexa Fluor secondary antibodies were used in this study, including Alexa Fluor 488 (green), Alexa Fluor 594 (red), and Alexa Fluor 647 (gray) (Life Technologies, Carlsbad, CA). Nuclei were identified by 4′,6-diamidino-2-phenylindole (DAPI) staining. Coverslips were mounted with ProLong gold antifade solution (Cell Signaling Technology). The images were acquired using Olympus FV1000 confocal microscopy systems and processed using FluoView software.

### HCMV attachment assay.

Increasing numbers of genome copies (10^4^, 10^5^, 10^6^, 10^7^, 10^8^) of WT or ΔUL132 HCMV were added to synchronized confluent HFFs prechilled to 4°C and allowed to attach at 4°C for 60 min. The cultures were then washed 4 times with PBS at 4°C. The cell-associated HCMV genome copy numbers remaining were then analyzed using qPCR after DNA extraction of the monolayer.

### HCMV entry assay.

Synchronized confluent HFFs were prechilled to 4°C and infected with equal genome copy numbers or equal MOIs of WT and ΔUL132 HCMV at 4°C. The cultures were incubated at 4°C for 60 min to allow virus attachment, shifted to 37°C for virus entry at different time points, and fixed in 4% PFA in PBS. Following washing and permeabilization, the monolayers were stained with antibodies against the HCMV tegument protein pp65 (mAb 28-19), and antibody binding was detected with an FITC-labeled anti-mouse IgG antibody. Infected HFFs with pp65 in the nucleus were quantified.

### Construction of lenti HFF cell lines.

A UL132-containing insert encoding amino acids 1 to 271 of the gpUL132 protein fused with an HA epitope was amplified with primers 5′-ACTGACTAGTATGCCGGCCCCGCG-3′ and 5′-ACTAGCGGCCGCCTAGTCGTACTCGGG-3′. The second insert, TrkBUL132, encoding the ectodomain and transmembrane domain from rat TrkB and the cytosol domain of the UL132 chimeric protein, was amplified with primers 5′-ACTAGAATTCGCCGCCACCATGTCGCC-3′ and 5′-GACTGGATCCTTAGTCGTACTCGGGATCTCTGAGC-3′. The inserts were cloned into the pLVX-puro vector to generate recombinant lentiviruses expressing the UL132 protein or the TrkBUL132 chimeric protein. Lentivirus-infected HFFs were selected under puromycin selection to produce lenti HFF cell lines stably expressing UL132 or TrkBUL132.
