# Supplementary figures and images for: Human Cytomegalovirus Envelope Protein gpUL132 Regulates Infectious Virus Production through Formation of the Viral Assembly Compartment
Source: mBio. 2020 Sep 29;11(5):e02044-20. doi: 10.1128/mBio.02044-20 (PMC7527726; doi:10.1128/mBio.02044-20)

Hu, et.al.

Supplementary figure 1.


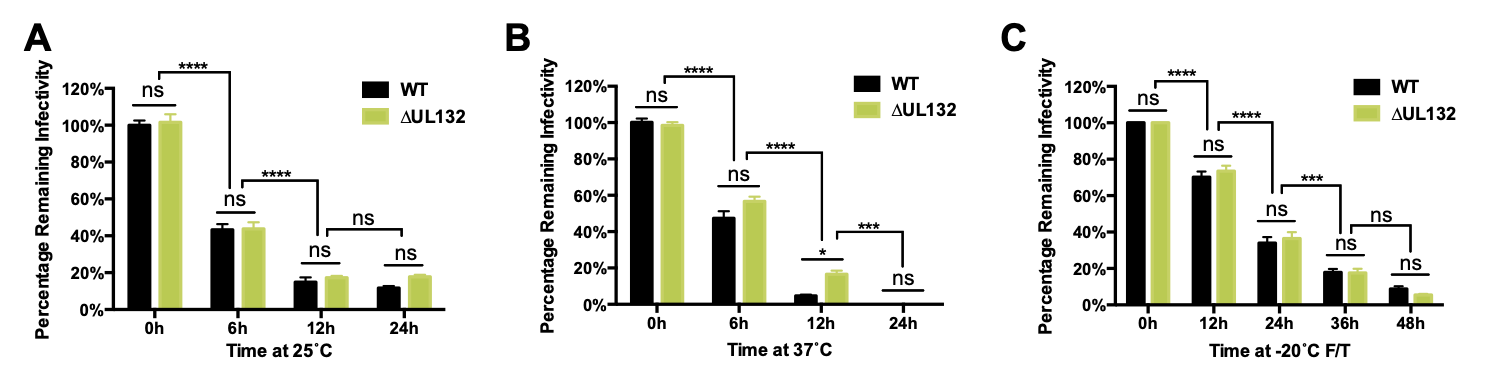

Supplement: FIG S1 [file mBio.02044-20-sf001.docx]

Wu, et.al.

Supplementary Figure 2


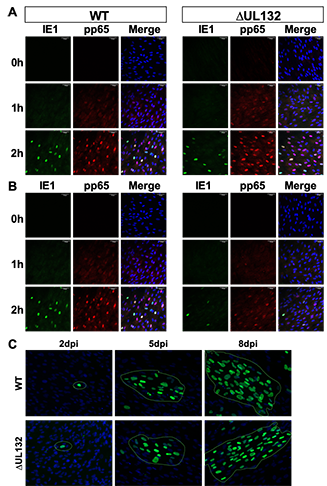


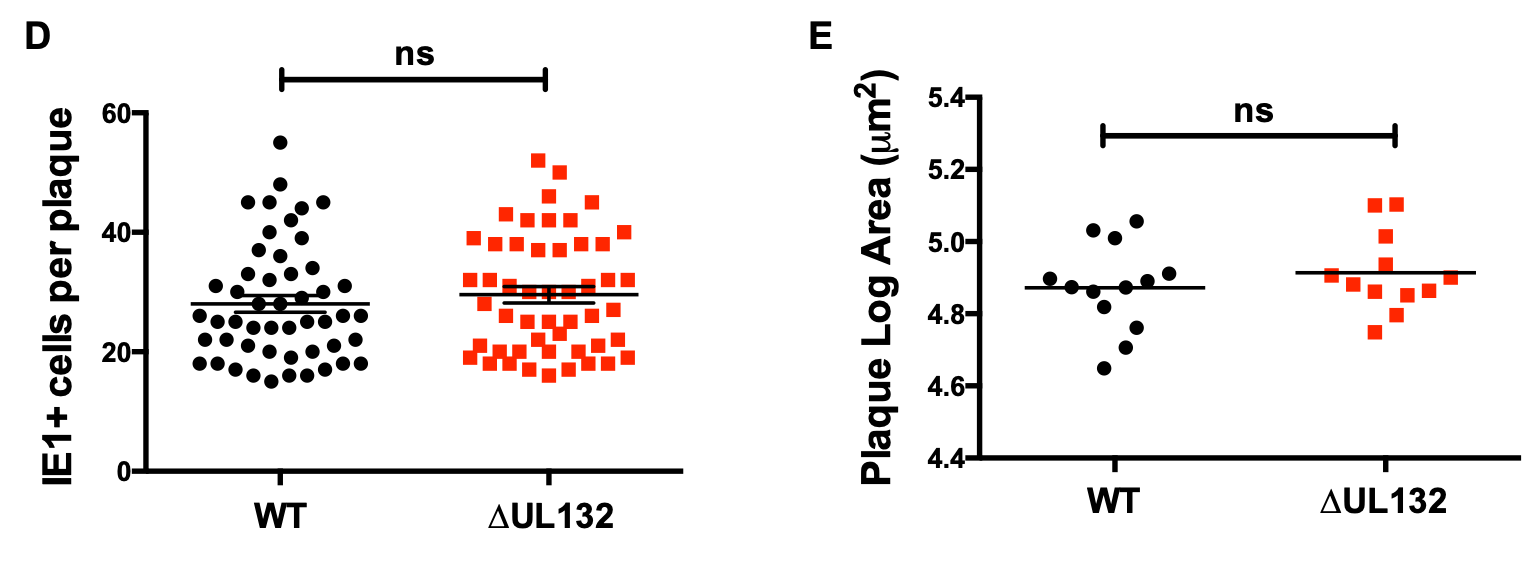

Supplement: FIG S2 [file mBio.02044-20-sf002.docx]

Wu, et.al.

Supplementary Figure 3


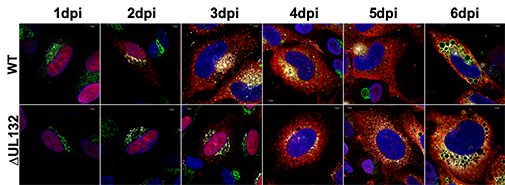

Supplement: FIG S3 [file mBio.02044-20-sf003.docx]
